# Folding of Truncated Granulin Peptides

**DOI:** 10.3390/biom10081152

**Published:** 2020-08-06

**Authors:** Rozita Takjoo, David Wilson, Paramjit S. Bansal, Alex Loukas, Michael J. Smout, Norelle L. Daly

**Affiliations:** Centre for Molecular Therapeutics, Australian Institute of Tropical Health and Medicine, James Cook University, Cairns, QLD 4870, Australia; rozita.takjoochelaras@my.jcu.edu.au (R.T.); david.wilson4@jcu.edu.au (D.W.); psoondh@hotmail.com (P.S.B.); alex.loukas@jcu.edu.au (A.L.)

**Keywords:** granulins, peptide, oxidative folding, NMR spectroscopy, cell proliferation

## Abstract

Granulins are a family of unique protein growth factors which are found in a range of species and have several bioactivities that include cell proliferation and wound healing. They typically contain six disulfide bonds, but the sequences, structures and bioactivities vary significantly. We have previously shown that an N-terminally truncated version of a granulin from the human liver fluke, *Opisthorchis viverrini*, can fold independently into a “mini-granulin” structure and has potent wound healing properties in vivo. The incorporation of a non-native third disulfide bond, with respect to the full-length granulin module, was critical for the formation of regular secondary structure in the liver fluke derived peptide. By contrast, this third disulfide bond is not required for a carp granulin-1 truncated peptide to fold independently. This distinction led us to explore granulins from the zebrafish model organism. Here we show that the mini-granulin fold occurs in a naturally occurring paragranulin (half-domain) from zebrafish, and is also present in a truncated form of a full-length zebrafish granulin, suggesting this structure might be a common property in either naturally occurring or engineered N-terminally truncated granulins and the carp granulin-1 folding is an anomaly. The in vitro folding yield is significantly higher in the naturally occurring paragranulin, but only the truncated zebrafish granulin peptide promoted the proliferation of fibroblasts consistent with a growth factor function, and therefore the function of the paragranulin remains unknown. These findings provide insight into the folding and evolution of granulin domains and might be useful in the elucidation of the structural features important for bioactivity to aid the design of more potent and stable analogues for the development of novel wound healing agents.

## 1. Introduction

**Figure 1:**
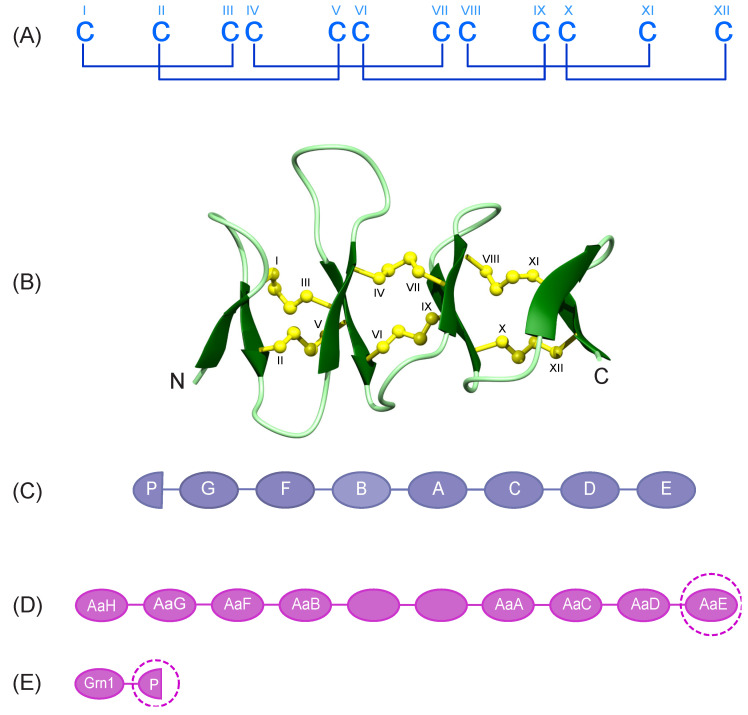
Granulin cysteine framework and precursor organization. (**A**) Schematic representation of the highly conserved cysteine framework and disulfide bond pairing present in the granulin family. The cysteine residues are numbered using Roman numerals (I-XII). (**Β**) The solution structure of full-length zebrafish granulin AaE (PDB ID: 6cku). Disulfide bonds are shown in yellow and cysteine residues are labelled using Roman numerals. (**C**) The order of the 7.5 granulin motifs in human progranulins, including A to G and a half-granulin motif named paragranulin (P) that includes the N-terminal six cysteines of the full length granulin. (**D**) Schematic representation of zebrafish progranulin A. Granulin modules with no label were missing from the partial clone [15]. (**E**) Granulin modules from zebrafish progranulin 1 including one full-length, and a half-granulin module named paragranulin (P) that comprises six cysteines of the full length granulin [15]. Target peptides of interest in the current study are shown with dashed circles.

Granulin proteins are prevalent throughout nature, being found in a wide variety of organisms [1]. They have potential as a novel class of therapeutic agents for treating a range of diseases and conditions, including chronic wounds [2,3,4,5,6,7,8,9,10,11]. Most granulins contain twelve cysteine residues, that form six disulfide bonds (Figure 1A,B), and are often expressed as progranulins containing multiple granulin modules. For example, the human and other mammalian granulin precursors (PGRN) contain a signal peptide and tandem repeats of seven-and-a-half granulin modules, as shown in Figure 1C for the human precursor [1,12]. Granulin G contains only ten cysteine residues and the half granulin module, paragranulin, comprises only the first six cysteine residues [13].

Significant sequence, structural and bioactivity variations exist across the granulin family [1,12]. The sequence conservation is primarily limited to the cysteine framework [12], with significant variation in the inter-cysteine loops. Structural diversity has been observed through the analysis of fish and human derived granulins. In contrast to the carp granulin-1 and zebrafish granulin AaE, which display relatively well-defined structures containing a four β-hairpin stack [14,15], several of the human granulin peptides display disordered regions. Human granulins A, C and F, for example, have well-defined N-terminal regions, but disordered C-terminal regions when produced recombinantly as single units, but it is unknown if this reflects the native granulin structures in biological systems. The bioactivity of the human granulins also varies, with granulin A shown to inhibit the proliferation of a breast cancer cell line, whereas human granulin F stimulates cell proliferation [16].

Truncated forms of granulins can fold into well-defined structures with only two or three disulfide bonds and, in some cases, have potent bioactivities. Peptides corresponding to the N-terminal region of carp granulin-1, an analogue of the N-terminal region of human granulin A (hGRNA) and a plant cysteine protease (oryzain β) analogue, can all fold independently into β-sheet structures with only two disulfide bonds [17,18,19,20]. However, we have recently shown that analogous peptides from the N-terminus of a granulin derived from the human liver fluke *Opisthorchis viverrini* (*Ov*-GRN-1) do not form a regular secondary structure [2,3]. By contrast, an *Ov*-GRN-1 derived peptide, (*Ov*-GRN_12-35_3s_), that contains three disulfide bonds, including a disulfide bond (CysIV-CysVI) not present in the full length granulins, can fold independently with a β-sheet structure. Interestingly, both the two and three-disulfide bond containing *Ov*-GRN-1 derived peptides display potent in vivo wound healing properties, similar to the full-length *Ov*-GRN-1 [2,3]. Indeed, an analogue of the *Ov*-GRN-1 peptide with three disulfide bonds has significant potential as a wound healing agent and is as potent as Regranex [2,3], a recombinant human platelet-derived growth factor, but can be made with synthetic methods rather than recombinant technologies. Regranex is currently the only approved biologic on the market for the treatment of chronic wounds [21,22].

The determination of whether granulins with sequences that differ significantly from *Ov*-GRN-1 can also fold independently while incorporating the CysIV-CysVI disulfide bond, is a critical step in advancing the understanding of the folding of this structural motif. Such an advance might have significant implications in the field of disulfide-rich peptide/protein structure and folding pathway predictions using molecular simulations [23]. On a more applied note, further understanding of the structure/function relationships of granulin-derived peptides might be useful in the design of wound healing agents for treating chronic wounds, such as diabetic foot ulcers.

To address the question regarding the folding of the N-terminal regions of granulins, we have analysed the structures of two truncated forms of zebrafish AaE, as well as a paragranulin from zebrafish progranulin 1. The peptides of interest in this study are highlighted with dashed circles in Figure 1D,E. In addition to a well-defined three-dimensional structure, zebrafish granulin AaE [15,24] promotes the survival of neuronal cells [15]. However, the structure or bioactivity of the half-module granulin from zebrafish has not been determined. Indeed, there have been limited studies on the naturally occurring paragranulins (half-modules). We show that incorporating a CysIV-CysVI disulfide bond into the N-terminal region of granulin might be a common feature across the family, but that the proliferative bioactivity of human cell lines is highly dependent on the primary structure.

## 2. Materials and Methods

### 2.1. Peptide Synthesis

The synthesis of truncated granulin peptides was undertaken by manual solid-phase peptide synthesis using standard 9-fluorenylmethyloxycarbonyl (Fmoc) chemistry. All peptides were assembled on 2-chlorotrityl chloride resin (Auspep, Tullamarine, Australia). The Fmoc amino acids (Auspep, Tullamarine, Australia) were activated using O-(1H-6-Chlorobenzotriazole-1-yl)-1,1,3,3-tetramethyluronium hexafluorophosphate, HCTU, (Iris Biotech GMBH, Marktredwitz, Germany) and coupled on the resin with DIPEA/DMF. Peptides were cleaved by a cleavage cocktail, including 95% trifluoracetic acid (TFA)/2.5% triisopropylsilane (TIPS)/2.5% H_2_O (*v*/*v*/*v*). TFA was removed with a stream of nitrogen gas. Peptides were then precipitated in ice-cold diethyl ether and dissolved in 50% acetonitrile: 50% H_2_O:0.1% TFA (*v*/*v*) and subsequently lyophilised. The peptides were synthesised without isotope labelling and the subsequent NMR spectra were recorded with natural abundance.

### 2.2. Purification

The crude peptides were purified using reversed-phase high performance liquid chromatography (RP-HPLC) on a C_18_ preparative column (Phenomenex Jupiter 250 × 21.2 mm, 10 μm, 300 Å) with gradients of solvent B (90% acetonitrile/10% H_2_O/0.045% TFA (*v*/*v*/*v*) and solvent A (H_2_O/0.05% TFA (*v*/*v*)). The masses of collected fractions were determined using a 5800 MALDI TOF/TOF mass spectrometer (SCIEX, Foster City, CA, USA).

### 2.3. Disulfide Bond Formation

The disulfide bonds of the truncated peptides were formed using non-regioselective oxidation in a solution of peptide in 0.1 M ammonium bicarbonate buffer (pH 8–8.2) and 5 mM reduced glutathione at room temperature. The oxidation reaction was left for 48 h prior to acidification with TFA (30–50 μL TFA to a 10 mL oxidation reaction) and loaded on a C_18_ preparative HPLC column with a flow rate of 6 mL/min. Fractions were collected and the peptide mass was analysed using a SCIEX 5800 MALDI TOF/TOF spectrometer [2,3] (Supplementary Figure S1).

### 2.4. NMR Spectroscopy

NMR samples were prepared from unlabelled purified peptide (0.2 mm) in 90% H_2_O/10% D_2_O. All NMR spectra were recorded on a 600 MHz AVANCE III NMR spectrometer (Bruker, Karlsruhe, Germany), equipped with a 5 mm TCI cryoprobe. Two-dimensional ^1^H−^1^H TOCSY, ^1^H−^1^H NOESY, ^1^H−^1^H DQF-COSY and ^1^H−^13^C HSQC spectra were acquired at 290 K. Spectra were recorded using an interscan delay of 1 s. NOESY spectra were acquired with mixing times of 200 ms, and TOCSY spectra were acquired with isotropic mixing periods of 80 ms. All spectra were processed using Bruker TopSpin (Version 3.5pl7) and assigned using CCPNMR analysis 2.1, based on the approach described in Wüthrich et al. [25,26]. More than 90% of the protons were assigned and 70% of the Cα and Cβ atoms were unambiguously assigned. Amide temperature coefficients were calculated based on TOCSY spectra directly referenced to DSS (4,4-dimethyl-4-silapentane-1-sulfonic acid) and recorded at temperatures ranging from 290 K to 305 K using the method outlined in Cierpicki et al. [27]. Hydrogen bond restraints (Supplementary Table S1) were included in the calculations based on hydrogen bonds identified in preliminary structures and amide protons with temperature coefficients more positive than −4.6 ppb/K.

The αH secondary shifts were determined by subtracting random coil ^1^H NMR chemical shifts from the experimental αH chemical shifts [28]. Root mean square deviation (RMSD) values were calculated relative to a mean structure using MOLMOL [29]. The structures and chemical shifts have been deposited into the Protein Data Bank and the Biological Magnetic Resonance Data Bank (ZF-N_24_3_—PDB ID: 7JIA, BMRB ID: 30780; ZF-para__3s_—PDB ID: 7JIY, BMRB ID: 30781).

### 2.5. Structure Calculations

The three-dimensional structures of peptides were calculated using the CYANA program, based on automated assignment of the NOEs [30]. Torsion-angle restraints predicted by TALOS-N were used in the structure calculations [31]. Structures were visualised using MOLMOL [29].

### 2.6. Mammalian Cell Culture

The 1BR.3.GN human skin normal fibroblast cell line was obtained from a European Collection of Authenticated Cell Cultures (ECACC, Porton Down, UK). The 1BR.3.GN cells were grown and maintained in Dulbecco’s Modified Eagle Medium/Nutrient Mixture F-12 (DMEM/F12) (Life Technologies, Melbourne, Australia) containing 1 × antibiotic/antimycotic and 1 × GlutaMAX, supplemented with 10% foetal bovine serum (FBS) (Gibco, Glasgow, Scotland) at 37 °C and 5% CO_2_. Cell proliferation assays were performed with DMEM/F12 media, supplemented with 10% FBS.

### 2.7. Cell Proliferation Monitoring in Real Time Using xCELLigence

Cells were seeded at 5000 cells/well in 170 μL of complete media in E-plates (ACEA Biosciences, San Diego, CA, United States) and grown overnight and monitored with an xCELLigence SP system (ACEA Biosciences), which monitors cellular events in real time by measuring electrical impedance across gold microelectrodes integrated into the base of tissue culture plates. Cells were washed three times with PBS prior to addition of 150 μL of low nutrient media and incubated for a minimum of 6 h before further treatment. Treatments were prepared at 8.5 × concentration and added to each well in a total volume of 20 μL. The xCELLigence system recorded cell indexes at intervals of 1 h for 5−6 days following treatment. Readings for the cell index were normalized prior to treatment, and cell proliferation ratios represent the relative numbers of cells compared to control cells at day 4. Comparisons of induction of cell proliferation in response to treatments were accomplished using the Two-Way ANOVA test with Dunnett’s multiple comparison correction, using GraphPad Prism 8.0.

## 3. Results

### 3.1. Design and Synthesis of Zebrafish Granulin Peptides

To analyse the folding of zebrafish granulins, three peptides were designed: ZF-N_24_2s,_ ZF-N_24_3s_ and ZF-para__3s_. The first peptide contains two disulfide bonds, and the latter two peptides contain three disulfide bonds. The sequences and sources of the synthetic peptides are shown in Table 1. An *Ov*-GRN-1 derived peptide is given in the table to highlight the differences in the inter-cysteine loop sequences.

**Figure 2:**
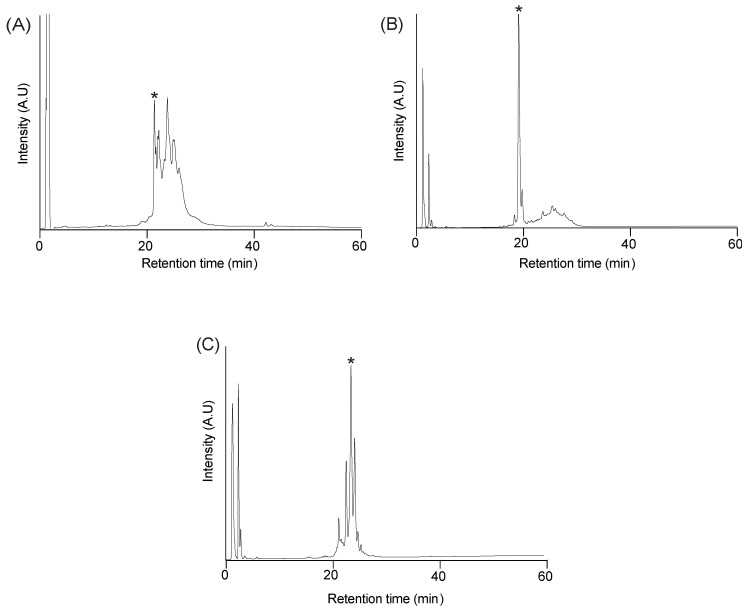
RP-HPLC analysis of the oxidation reaction of granulin N-terminal truncated analogues (**A**) ZF-N_24_3s_, (**B**) ZF-para__3s_ and (**C**) ZF-N_24_2s_. Analytical RP-HPLC was carried out using a Phenomenex Jupiter 4 µm Proteo column (C_12_, 150 × 2.00 mm, 10 μm, 90 Å) using a gradient of 0–60% solvent Β (Solvent A: 99.95% H_2_O: 0.05% TFA; Solvent Β: 90% acetonitrile: 10% H_2_O: 0.045% TFA) over 60 min with a flow rate of 0.4 mL/min. The absorbance was monitored at 214 nm. The early eluting sharp peaks (highlighted by asterisks (*)) were purified for subsequent analyses.

Peptides were synthesised using Fmoc chemistry on 2-chlorotrityl resin without selective protection of the cysteine residues, and the disulfide bonds formed by air oxidation in ammonium bicarbonate (0.1 M) and reduced glutathione (5 mM) at room temperature. The analytical RP-HPLC trace of all three oxidation reactions contain several peaks, including a sharp peak that eluted earlier than the other peaks and corresponds to a fully oxidised isomer, based on mass spectrometry. The early eluting peaks were purified (>95% purity based on analytical RP-HPLC) and the structures analysed using NMR spectroscopy. The yields of the early eluting peaks varied amongst the peptides. A comparison of the folding of the peptides is shown in Figure 2, highlighting the higher yield obtained for the naturally occurring paragranulin compared to the truncated version of zebrafish granulin AaE (ZF-N_24_3s_).

### 3.2. Structural Analysis with NMR Spectroscopy

**Figure 3:**
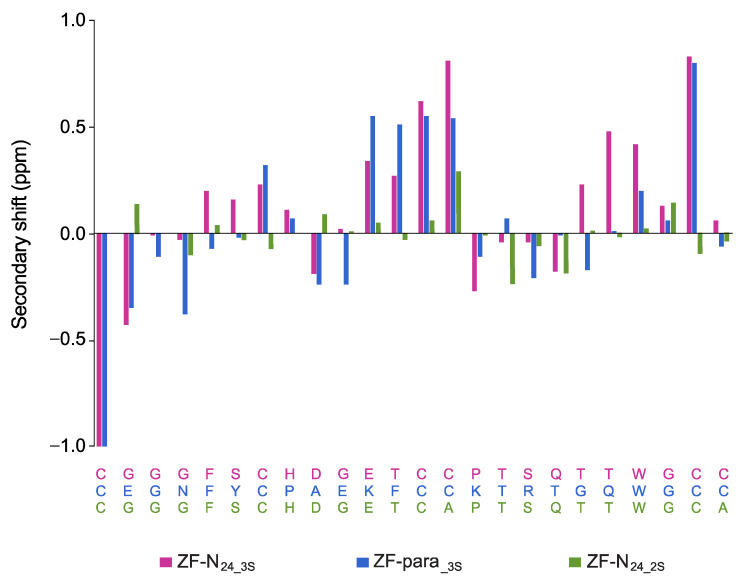
αH secondary-shift comparison for truncated granulin analogues. The αH secondary shifts were calculated by subtracting the random coil ^1^H NMR chemical shifts previously reported by Wishart et al. [28] from the experimental αH chemical shifts. The sequences of ZF-N_24_3s_ (violet), ZF-para__3s_ (blue) and ZF-N_24_2s_ (green) are given at the bottom of the diagram and are colour coded with respect to the graph.

The one-dimensional proton NMR spectra of ZF-N_24_3s_ and ZF-para__3s_ display large chemical shift dispersion in the amide region, and analysis of the secondary shifts indicates that the peptides contain β-sheet structure based on the consecutive positive shifts (Figure 3). The three-dimensional structures of both peptides were calculated using CYANA based on distance and dihedral angle restraints derived from one- and two-dimensional homonuclear and heteronuclear NMR experiments. Calculation of the structures without disulfide bond restraints indicated that the most likely connectivity was CysI-CysIII, CysII-CysV and CysIV-CysVI, consistent with the previous study on *Ov*-GRN-1 peptides [2,3]. Calculation of the structures with all 15 possible disulfide bond connectivities also indicated that this connectivity had the lowest target function (Supplementary Table S2). Subsequent structures were calculated using restraints for this disulfide connectivity. Hydrogen bond restraints were included based on analysis of the preliminary structures and temperature coefficients (Supplementary Tables S1, S3 and S4). The refinement statistics are given in Table 2.

In contrast to the three-disulfide bond containing peptides, ZF-N_24_2s_ did not display significant dispersion in the amide region of the one-dimensional proton spectrum. Despite this lack of dispersion in the amide region, the backbone protons and most of the side chain protons could be assigned based on two-dimensional spectra. Analysis of the secondary shifts (Figure 3) indicates that the peptide does not contain regular secondary structure, as the majority of the shifts are within 0.1 ppm of the random coil values.

The major element of secondary structure in ZF-N_24_3s_ and ZF-para__3s_ is a β-hairpin comprising residues 13–23. An NOE between the α-protons of Cys 13 and Cys 23 is clearly present in both spectra, and represents a key restraint in defining the alignment of the β-strands. The structures appear to be stabilised by hydrogen bonds consistent with amide protons, having temperature coefficients more positive than −4.6 ppb/K [27]. The disulfide bonds have the connectivity CysI-CysIII, CysII-CysV and CysIV-CysVI; the two former disulfide bonds are equivalent to those present in the full-length structures of the well characterised granulins [14,18]. In the full-length structures CysIV is bonded to CysVII and CysVI is bonded to CysIX (refer to Figure 1A for disulfide bond connectivity) [15,16,32].

**Figure 4:**
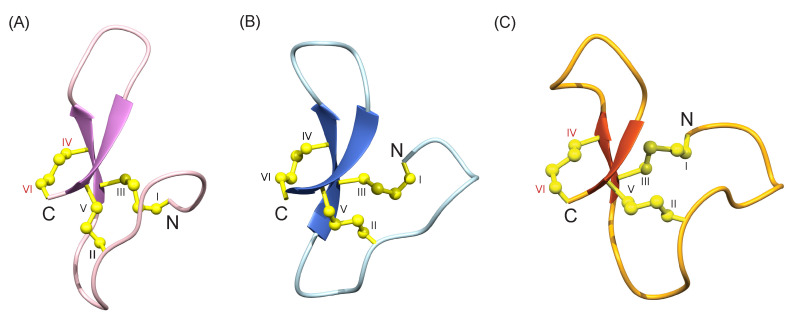
Three-dimensional structures of (**A**) ZF-N_24_3s_, (**B**) ZF-para__3s_ and (**C**) *Ov*-GRN_12-35_3s_ [2] N-terminal truncated peptides. The β-hairpins are shown as arrows, and ZF-N_24_3s_ is shown in violet, ZF-para__3s_ in blue and *Ov*-GRN_12-35_3s_ (PDB: 5UJG) in orange. Disulfide bonds are shown in yellow and cysteine residues are labelled using Roman numerals. The cysteine residues involved in forming the disulfide bond (IV-VI) not present in the full-length granulin structures of zebrafish granulin AaE and by homology *Ov*-GRN-1, are labelled with red letters (**A**,**C**). The figure was prepared using MOLMOL [29].

**Figure 5:**
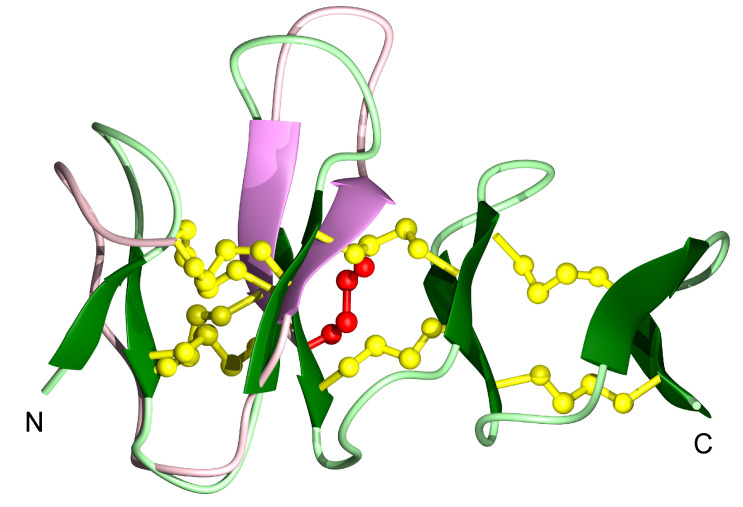
Superposition of the structures of the ZF-N_24-3s_ and zebrafish AaE. The β-hairpins are shown as arrows—in violet for ZF-N_24-3s_ (PDB ID: 7JIA), and dark green for zebrafish AaE (PDB ID: 6cku). Disulfide bonds are shown in yellow. The cysteine residues involved in forming the non-native disulfide bond (IV-VI) in ZF-N_24-3s_ is shown in red. The figure was prepared using MOLMOL [29].

Comparison of the structures of ZF-N_24_3s_ and ZF-para__3s_ with the structure of a truncated form of *Ov*-GRN-1 (*Ov*-GRN_12-35_3s_) [2,3], also containing the first six cysteine residues, indicates the peptides have similar overall folds despite the sequence differences (37.5% sequence identity to *Ov*-GRN_12-35_3s_; the sequence identity is primarily related to the conserved cysteine residues) (Figure 4). A superposition of ZF-N_24_3s_ with the structure of zebrafish granulin AaE is given in Figure 5 and highlights the similarity between the truncated and full-length versions, despite the differences in disulfide bonds.

### 3.3. Cell Proliferation Assay

**Figure 6:**
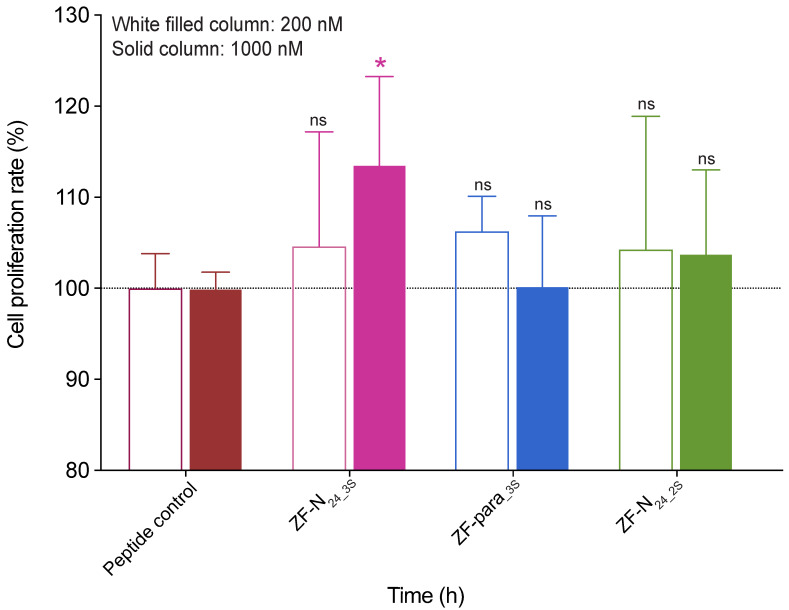
Cell proliferation analysis of granulin peptides using xCELLigence technology. Fibroblast cells were treated with peptides at concentrations of 200 nM and 1 μM. Data represent mean ± SD of three independent experiments. Data were analysed by one-way ANOVA against peptide control: ns—not significant; ∗—*p* < 0.05. Cell index was measured at day 4 after treatment. White-filled columns and solid columns represent concentrations of 200 nM and 1 μM, respectively.

The effect of the truncated peptides on the growth of 1BR.3.GN fibroblasts was evaluated using an xCELLigence system. The 1BR.3.GN is a human fibroblast skin cell line derived from transformed normal fibroblasts. Cells were cultured with two concentrations of the peptides, 200 nM and 1 μM. ZF-N_24_3s_ significantly promoted the proliferation of fibroblasts compared to the negative control peptide (a 19-residue peptide from tropomyosin) (Figure 6). The proliferation rate for ZF-N_24_3_ was 113.6% (*p* < 0.05), relative to the control peptide at day 4 after treatment. By contrast, the effect of ZF-para_-3s_ or ZF-N_24_2S_ on fibroblast cell proliferation was not statistically significant at either 200 nM or 1 μM (Figure 6).

## 4. Discussion

Folding of disulfide rich peptides still represents one of the great challenges in structure prediction and simulation [33,34]. Experimental analysis of the folding processes has to underlie these simulations, and analysis of the granulin structural framework present in the vast majority of organisms is likely to provide valuable insight for future computational studies. The potential as a wound healing agent might also be enhanced as a result of greater understanding of the structure/function relationships. In the current study we show that the N-terminal half of a range of granulins can fold independently of the C-terminal region via the incorporation of a CysIV-CysVI disulfide bond, indicating this is a common feature in this family. It appears likely that the naturally occurring half-motifs are optimised for folding compared to engineered versions derived from full-length granulin sequences, and that the CysIV-CysVI disulfide bond likely constitutes a native disulfide bond. However, the structure of a paragranulin on native material will be required to confirm this suggestion.

The secondary shifts of the granulin peptides ZF-N_24_3s_ and ZF-para__3s_ were similar (Figure 3), indicating they have similar structures, and this was subsequently confirmed by the determination of the three-dimensional structures of the peptides (Figure 4). Both peptides contain a β-hairpin and similar arrangement of the disulfide bonds to *Ov*-GRN_12-35_3s_ [2,3], the first granulin peptide shown to adopt this conformation with three disulfide bonds. The common fold we have identified for the half-granulin domain could represent an evolutionary ancestor of the full-length scaffold, hence the propensity for a range of granulins to adopt this fold in the truncated form even though the disulfide connectivity differs in the full-length proteins.

The more efficient in vitro folding of the paragranulin-derived peptide, ZF-para__3s_, relative to the engineered version (ZF-N_24_3S_), poses questions regarding the sequence differences between the peptides and the subsequent folding pathways. β-hairpins can represent the folding nucleus with hydrophobic collapse being an integral part of folding [35]. However, comparison of the sequences of the two zebrafish peptides indicates very little difference in the hydrophobicity of the amino acids involved in the β-hairpin loop and therefore it is difficult to speculate the reason for the folding differences.

The structural fold present in these truncated granulin peptides has recently been referred to as the “mini-granulin” fold and is present in several peptides [36], including conotoxins ϕ-MiXXVIIA [37] and H-Vc7.2. The fold contains two conserved disulfide bonds, which correspond to CysI-CysIII and CysII-CysV in ZF-N_24_3s_ and ZF-para__3s_. Both ϕ-MiXXVIIA and H-Vc7.2 contain additional disulfide bonds, but they are distinct from the additional CysIV-CysVI bond present in ZF-N_24_3s_ and ZF-para__3s._ However, there are examples of this motif containing only two disulfide bonds, such as the truncated form of carp granulin-1, which forms a well-defined β-sheet-containing structure [16,17], indicating that the mini-granulin fold is not always dependent on additional disulfide bonds.

The β-sheet structure present in the carp granulin-1 truncated peptide is in contrast to the results obtained in the current study on ZF-N_24_2s_, which has no regular secondary structure. Previous studies have shown that, when only CysI-CysIII and CysII-CysV are present in a truncated form of human granulin A, the peptide does not form a well-defined structure [17]. Similarly, a truncated form of *Ov*-GRN-1 with only two disulfide bonds does not form β-sheet structure, whereas the three-disulfide bond form does [2,3]. The results to date suggest that the folding of the carp granulin-1 peptide could be an anomaly, but further study is required to determine residues required for effective folding.

In addition to providing insight into granulin folding, the current study also analysed the biological activity of the truncated N-terminal peptides. ZF-N_24_3s_ displayed activity in the cell proliferation assay, whereas ZF-para__3s_ was not active. However, ZF-N_24_3s_ was not as active as the parasite-derived granulin peptide *Ov*-GRN_12−35_3s_ [2]. The cells chosen for study in the current study reflect the potential of granulin peptides as wound healing agents, but it is possible the zebrafish derived peptides would show greater potency on fish-derived cell lines. There are now several examples where it has been shown that well-defined 3D structures are not necessary for bioactivity [38], including human granulin Β, which has been characterised as an intrinsically disordered protein that modestly induces NF-κB activation in SY-SH5Y human neuroblastoma cells [39]. The current study highlights the importance of the primary structure, given that all peptides contain a β-hairpin, but the activity varies significantly.

## 5. Conclusions

Overall, this study has shown that truncated granulins can incorporate a disulfide bond not-present in the full-length module, and this bond plays a pivotal role in stabilizing a well-defined structure in the N-terminal region of truncated granulin peptides. Despite a common structural motif, the bioactivity of these engineered peptides varies, and further mutational studies will be required to determine important regions/residues for bioactivity. Ultimately, the identification of the molecular target is likely to provide the greatest advance in understanding the structure/function relationships of granulin peptides.

## Figures and Tables

**Table 1:** Granulin-derived peptide sequences.

Peptide	Source	Sequence
ZF-N_24_3s_	Zebrafish granulin AaE	**C** GGGF-S **C** HDGET **C** **C** PTSQTTWG **C** **C**
ZF-N_24_2s_	Zebrafish granulin AaE	**C** GGGF-S **C** HDGET **C** APTSQTTWG **C** A
ZF-para__3s_	Zebrafish paragranulin	**C** EGNFY- **C** PAEKF **C** **C** KTRTGQWG **C** **C**
*Ov*-GRN_12-35_3s_^#^	*Ov*-GRN-1	**C** PDPVYT **C** RPGQT **C** **C** RGLHG-YG **C** **C**

Hyphen (–) indicates a sequence gap. Cysteine residues are shown in bold and red. ^#^ The sequence of the previously studied *Ov*-GRN_12-35_3s_ [2], is cited accordingly and presented in this table for comparison purposes. The sequences of the peptides synthesised in the current study are highlighted in blue. The cysteine residues involved in the non-native (CysIV-CysVI) disulfide bond in zebrafish granulin AaE, and by homology in *Ov*-GRN-1 (the 3D structure of *Ov*-GRN-1 has not been determined), have been underlined. The sequences are derived from the precursor proteins of zebrafish granulin A (Q90ZD0), zebrafish progranulin-1 (Q8QGN9) and *Ov*-GRN-1 (B8XSI4).

**Table 2:** Structural statistics.

Experimental Restraints	ZF-N_24_3s_	ZF-para__3s_
Interproton distance restraints		
*Intra-residue, |i − j| = 0*	41	72
*Sequential, |i − j| = 1*	65	88
*Medium range, 1 < |i − j| < 5*	12	36
*Long range, |i − j| > = 5*	31	64
Disulfide-bond restraints (3 restraints per bond)	9	9
Dihedral-angle restraints	27	32
Hydrogen bond restraints (2 restraints per bond)	4	8
**Root Mean Square Deviations from Mean Coordinate Structure (Å)**		
Backbone atoms	1.29 ± 0.38	0.63 ± 0.20
All heavy atoms	1.81 ± 0.41	1.31 ± 0.35
**Ramachandran Statistics**		
% in most favoured region	80.6	96.4
% Residues in additionally allowed regions	19.4	3.6

## Supplementary Materials

The following are available online at https://www.mdpi.com/2218-273X/10/8/1152/s1, Figure S1: SCIEX TOF/TOF™ 5800 MALDI mass spectra of ZF-N_24_3s_, ZF-para__3s_ and ZF-N_24_2s_ using α-cyano-4- hydroxycinnamic acid (CHCA) matrix. The spectra in panels A, B and C correspond to the reduced peptides and the spectra in panels D, E and F correspond to the oxidised peptides. The masses of reduced and oxidised peptides are highlighted by asterisks (*), Table S1: Hydrogen bond restraints, Table S2: Cyana target functions for all 15 possible disulfide bond connectivities for each peptide, Table S3: Temperature coefficients for ZF-N_24_3s_, Table S4: Temperature coefficients for ZF-para__3s_.

## References

[1] Palfree R.G., Bennett H.P., Bateman A. (2015). The evolution of the secreted regulatory protein progranulin. PLoS ONE.

[2] Bansal P.S., Smout M.J., Wilson D., Caceres C.C., Dastpeyman M., Sotillo J., Seifert J., Brindley P.J., Loukas A., Daly N.L. (2017). Development of a potent wound healing agent based on the liver fluke granulin structural fold. J. Med. Chem..

[3] Dastpeyman M., Bansal P.S., Wilson D., Sotillo J., Brindley P.J., Loukas A., Smout M.J., Daly N.L. (2018). Structural variants of a liver fluke derived granulin peptide potently stimulate wound healing. J. Med. Chem..

[4] Smout M.J., Sotillo J., Laha T., Papatpremsiri A., Rinaldi G., Pimenta R.N., Chan L.Y., Johnson M.S., Turnbull L., Whitchurch C.B. (2015). Carcinogenic parasite secretes growth factor that accelerates wound healing and potentially promotes neoplasia. PLoS Pathog..

[5] Botelho M.C., Alves H., Richter J. (2016). Wound healing and cancer progression in Opisthorchis viverrini associated cholangiocarcinoma. Parasitol. Res..

[6] Ding H., Wei J., Zhao Y., Liu Y., Liu L., Cheng L. (2017). Progranulin derived engineered protein Atsttrin suppresses TNF-α-mediated inflammation in intervertebral disc degenerative disease. Oncotarget.

[7] Qiao G., Xu H.L., Li C., Li X., Farooqi A.A., Zhao Y.M., Liu X.H., Liu M., Stagos D., Lin X.K. (2018). Granulin A synergizes with cisplatin to inhibit the growth of human hepatocellular carcinoma. Int. J. Mol. Sci..

[8] Chitramuthu B.P., Bennett H.P.J., Bateman A. (2017). Progranulin: A new avenue towards the understanding and treatment of neurodegenerative disease. Brain.

[9] Jian J., Tian Q.-Y., Hettinghouse A., Zhao S., Liu H., Liu C.-J., Wei J., Grunig G., Zhang W., Setchell K.D.R. (2016). Progranulin recruits HSP70 to β-glucocerebrosidase and is therapeutic against Gaucher disease. EBioMedicine.

[10] Abella V., Pino J., Scotece M., Conde J., Lagoa F., Gonzalez-Gay M.A., Mera A., Gomez R., Mobasheri A., Gualillol O. (2017). Progranulin as a biomaker and potential therapeutic agent. Drug Discov. Today.

[11] Pogonowska M., Poniatowski Ł.A., Wawrzyniak A., Królikowska K., Kalicki B. (2019). The role of progranulin (PGRN) in the modulation of anti-inflammatory response in asthma. Cent. Eur. J. Immunol..

[12] Dastpeyman M., Smout M.J., Wilson D., Loukas A., Daly N.L. (2018). Folding of granulin domains. Pept. Sci..

[13] Ong C.H.P., Bateman A. (2003). Progranulin (granulin-epithelin precursor, PC-cell derived growth factor, acrogranin) in proliferation and tumorigenesis. Histol. Histopathol..

[14] Hrabal R., Chen Z., James S., Bennett H.P.J., Ni F. (1996). The hairpin stack fold, a novel protein architecture for a new family of protein growth factors. Nat. Struct. Biol..

[15] Wang P., Chitramuthu B., Bateman A., Bennett H.P.J., Xu P., Ni F. (2018). Structure dissection of zebrafish progranulins identifies a well-folded granulin/epithelin module protein with pro-cell survival activities: Dynamic structures of zebrafish granulin modules. Protein Sci..

[16] Tolkatchev D., Malik S., Vinogradova A., Wang P., Chen Z., Xu P., Bennett H.P.J., Bateman A., Ni F. (2008). Structure dissection of human progranulin identifies well-folded granulin/epithelin modules with unique functional activities. Protein Sci..

[17] Tolkatchev D., Ng A., Vranken W., Ni F. (2000). Design and solution structure of a well-folded stack of two β-hairpins based on the amino-terminal fragment of human granulin A. Biochemistry.

[18] Vranken W.F., Chen Z.G., Xu P., James S., Bennett H.P.J., Ni F. (1999). A 30-residue fragment of the carp granulin-1 protein folds into a stack of two β-hairpins similar to that found in the native protein. J. Pept. Res..

[19] Vranken W.F., James S., Bennett H.P.J., Ni F. (2002). Solution structures of a 30-residue amino-terminal domain of the carp granulin-1 protein and its amino-terminally truncated 3-30 subfragment: Implications for the conformational stability of the stack of two β-hairpins. Proteins.

[20] Tolkatchev D., Xu P., Ni F. (2001). A peptide derived from the C-terminal part of a plant cysteine protease folds into a stack of two β-hairpins, a scaffold present in the emerging family of granulin-like growth factors. J. Pept. Res..

[21] Niezgoda J.A., Van Gils C.C., Frykberg R.G., Hodde J.P. (2005). Randomized clinical trial comparing OASIS Wound Matrix to Regranex Gel for diabetic ulcers. Adv. Skin Wound Care.

[22] Chan R.K., Liu P.H., Pietramaggiori G., Ibrahim S.I., Hechtman H.B., Orgill D.P. (2006). Effect of recombinant platelet-derived growth factor (Regranex^®^) on wound closure in genetically diabetic mice. J. Burn Care Res..

[23] Georgoulia P.S., Glykos N.M. (2019). Molecular simulation of peptides coming of age: Accurate prediction of folding, dynamics and structures. Arch. Biochem. Biophys..

[24] Wang P.F. (2004). Structure Genomics of Zebrafish Granulins.

[25] Wüthrich K. (2003). NMR studies of structure and function of biological macromolecules (Nobel Lecture). J. Biomol. NMR.

[26] Vranken W.F., Boucher W., Stevens T.J., Fogh R.H., Pajon A., Llinas M., Ulrich E.L., Markley J.L., Ionides J., Laue E.D. (2005). The CCPN data model for NMR spectroscopy: Development of a software pipeline. Proteins.

[27] Cierpicki T., Otlewski J. (2001). Amide proton temperature coefficients as hydrogen bond indicators in proteins. J. Biomol. NMR.

[28] Wishart D.S., Bigam C.G., Holm A., Hodges R.S., Sykes B.D. (1995). ^1^H, ^13^C and ^15^N random coil NMR chemical shifts of the common amino acids. I. Investigations of nearest-neighbor effects. J. Biomol. NMR.

[29] Koradi R., Billeter M., Wüthrich K. (1996). MOLMOL: A program for display and analysis of macromolecular structures. J. Mol. Graph..

[30] Güntert P. (2004). Automated NMR structure calculation with CYANA. Methods Mol. Biol..

[31] Shen Y., Bax A. (2015). Protein structural information derived from NMR chemical shift with the neural network program TALOS-N. Methods Mol. Biol..

[32] Bhandari V., Roger G.E.P., Bateman A. (1992). Isolation and sequence of the granulin precursor cDNA from human bone marrow reveals tandem cysteine-rich granulin domains. Proc. Natl. Acad. Sci. USA.

[33] Yang J., He B.-J., Jang R., Zhang Y., Shen H.-B. (2015). Accurate disulfide-bonding network predictions improve ab initio structure prediction of cysteine-rich proteins. Bioinformatics.

[34] Paul George A.A., Heimer P., Maaß A., Hamaekers J., Hofmann-Apitius M., Biswas A., Imhof D. (2018). Insights into the folding of disulfide-rich μ-conotoxins. ACS Omega.

[35] Lewandowska A., Ołdziej S., Liwo A., Scheraga H.A. (2010). β-hairpin-forming peptides; models of early stages of protein folding. Biophys. Chem..

[36] Nielsen L.D., Foged M.M., Albert A., Bertelsen A.B., Søltoft C.L., Robinson S.D., Petersen S.V., Purcell A.W., Olivera B.M., Norton R.S. (2019). The three-dimensional structure of an H-superfamily conotoxin reveals a granulin fold arising from a common ICK cysteine framework. J. Biol. Chem..

[37] Jin A.H., Dekan Z., Smout M.J., Wilson D., Dutertre S., Vetter I., Lewis R.J., Loukas A., Daly N.L., Alewood P.F. (2017). Conotoxin ϕ-MiXXVIIA from the superfamily G2 employs a novel cysteine framework that mimics granulin and displays anti-apoptotic activity. Angew. Chem. Int. Ed..

[38] Uversky V.N. (2013). A decade and a half of protein intrinsic disorder: Biology still waits for physics. Protein Sci..

[39] Ghag G., Wolf L.M., Reed R.G., Van Der Munnik N.P., Mundoma C., Moss M.A., Rangachari V. (2016). Fully reduced granulin-B is intrinsically disordered and displays concentration-dependent dynamics. Protein Eng. Des. Sel..

