# Supplementary material for: Folding of Truncated Granulin Peptides
Source: Biomolecules. 2020 Aug 6;10(8):1152. doi: 10.3390/biom10081152 (PMC7463432; doi:10.3390/biom10081152)
Supplement: Supplementary file 1 [file biomolecules-10-01152-s001.pdf]

Supplementary Materials:

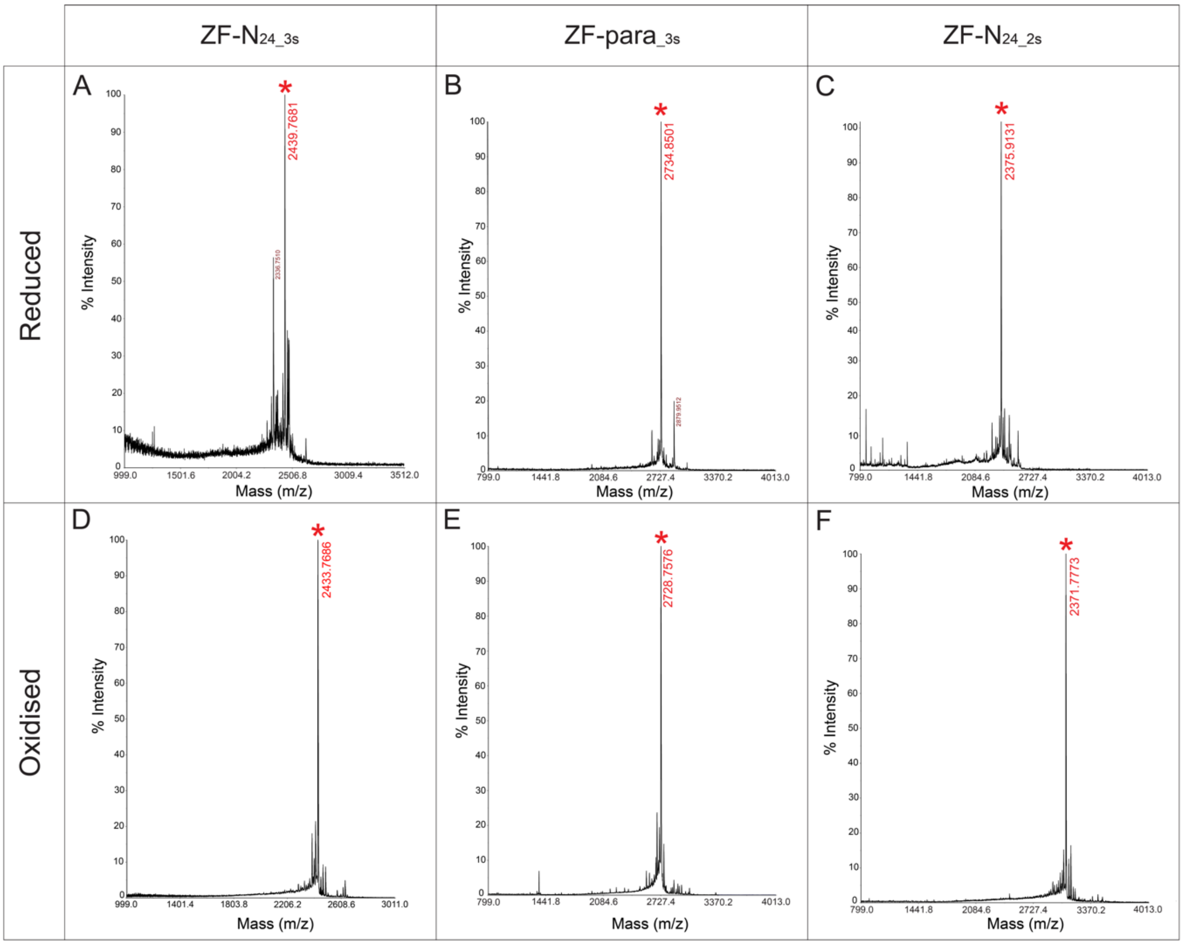

Figure S1. SCIEX TOF/TOF™ 5800 MALDI mass spectra of ZF-N$_{24_3s}$, ZF-para$_{_3s}$ and ZF-N$_{24_2s}$ using α-cyano-4-hydroxycinnamic acid (CHCA) matrix. The spectra in panels A, B and C correspond to the reduced peptides and the spectra in panels D, E and F correspond to the oxidised peptides. The masses of reduced and oxidised peptides are highlighted by asterisks (*).

Table S1. Hydrogen bond restraints.

ZF-N$_{24_3s}$	ZF-para$_{_3s}$
11 GLU HN 8 HIS O	14 CYS HN 22 GLY O
17 SER HN 20 THR O	16 THR HN 20 GLN O
	24 CYS HN 12 THR O
	19 GLY HN 16 THR O

Table S2. Cyana target functions for all 15 possible disulfide bond connectivities for each peptide.

		Target Functions	
	**Connectivity**	**ZF-N$_{24_3s}$**	**ZF-para$_{_3s}$**
1	1-13, 7-23, 14-24	0.0254 ± 0.00303	0.10 ± 0.000367
2	1-13, 7-14, 23-24	1.42 ± 0.20	1.74 ± 0.0151
3	1-13, 7-24, 14-23	0.10 ± 0.0397	0.43 ± 0.15
4	1-7, 14-24, 13-23	0.0683 ± 0.0205	0.28 ± 0.0179
5	1-7, 13-24, 14-23	0.0741 ± 0.0228	0.51 ± 0.0359
6	1-7, 13-14, 23-24	4.82 ± 0.00338	10.44 ± 0.0497
7	1-14, 7-13, 23-24	0.99 ± 0.00248	1.11 ± 0.00313
8	1-14, 7-23, 13-24	0.0542 ± 0.0177	3.55 ± 0.0785
9	1-14, 7-24, 13-23	0.11 ± 0.0587	0.17 ± 0.0497
10	1-23, 7-13, 14-24	0.0310 ± 0.00613	0.28 ± 0.0592
11	1-23, 7-14, 13-24	0.31 ± 0.10	1.19 ± 0.0178
12	1-23, 7-24, 13-14	3.94 ± 0.0457	9.36 ± 0.0445
13	1-24, 7-13, 14-23	0.0584 ± 0.0257	0.65 ± 0.0273
14	1-24, 7-14, 13-23	0.68 ± 0.23	1.26 ± 0.0377
15	1-24, 7-23, 13-14	3.88 ± 0.0353	9.12 ± 0.0118

The numbers in red indicate the connectivity with the lowest target function for each peptide.

Table S3. Temperature coefficients for ZF-N$_{24_3s}$.

**Residue**	**−Δδ$_{NH}$/ΔT (ppb/K)**
11-Glu	−1.584
17-Ser	−1.124

Table S4. Temperature coefficients for ZF-para$_{_3s}$.

**Residue**	**-Δδ$_{NH}$/ΔT (ppb/K)**
24-Cys	-3.204
14-Cys	-4.052
16-Thr	-4.048
19-Gly	-1.844
18-Thr	-1.962
20-Gln	-3.006
